# P-1380. Congenital Syphilis at Los Angeles General Medical Center, 2022-2023

**DOI:** 10.1093/ofid/ofae631.1556

**Published:** 2025-01-29

**Authors:** Diana Villarreal, Katherine Lewis, Mikhaela Cielo, Jeffrey Klausner

**Affiliations:** Los Angeles General Medical Center, University of Southern California, Glendale, California; University of Southern California, Los Angeles, California; Los Angeles General Medical Center/USC, Los Angeles, California; Keck School of Medicine of USC, Los Angeles, California

## Abstract

**Background:**

Congenital syphilis cases in the United States have increased 10-fold in the last decade. At Los Angeles General Medical Center (LAGMC), a large public hospital in Los Angeles, infants born to mothers with untreated syphilis during pregnancy continue to rise. We reviewed all the cases of neonatal congenital syphilis in 2022 and 2023 to describe sociodemographic and clinical characteristics and management history.

**Methods:**

A search of the LAGMC Electronic Health Records uncovered all infant RPR test results in 2022 and 2023. Once case-patients were identified, we abstracted relevant information related to maternal health and infection history along with the infant medical history into a Redcap database. Statistical analysis was performed.

**Results:**

Thirty-six infants were born to mothers with a history of inadequately treated syphilis in pregnancy. Compared to women who were adequately treated before or during pregnancy (n=62), women that were not adequately treated were more likely to have methamphetamine use (72% vs. 41%, p=0.004), cocaine use (19% vs. 2%, p=0.005), opiate use (34% vs. 9%, p=0.002), or no prenatal care (41% vs. 8%, p ≤ 0.001). Of the 12 infants with probable congenital syphilis, two infants had an abnormal physical exam. One infant had an RPR > 4-fold the mother’s RPR. Four infants had bony changes on long bone radiography. Of the 36 exposed infants, 20 had successful lumbar punctures. Five met criteria for probable congenital syphilis from elevated CSF protein alone, with normal cell counts and non-reactive VDRL. Two infants had positive CSF VDRL.

**Conclusion:**

Overall, 2 infants out of 12 with probable congenital syphilis were symptomatic at the time of birth, and the other 10 were identified from laboratory and radiographic evaluation. Sixteen infants did not have a successful lumbar puncture, but results of the lumbar puncture did not change treatment choice. Our review also identified maternal substance use and no prenatal care as risk factors for inadequate treatment of syphilis before or during pregnancy.

**Disclosures:**

**Jeffrey Klausner, MD MPH**, Direct Diagnostics: Advisor/Consultant

